# A Rare Case of Tuberculous Osteomyelitis in a Toddler: Diagnostic Clues and Management Approach

**DOI:** 10.7759/cureus.89235

**Published:** 2025-08-01

**Authors:** Karthik Prakash, Prabhakaran A, Praveen Iyappan, Ashok T Ramanujam

**Affiliations:** 1 Orthopaedics, Mahatma Gandhi Medical College and Research Institute, Pondicherry, IND

**Keywords:** extrapulmonary tuberculosis (eptb), osteomyelitis, skeletal tuberculosis, toddler, tuberculosis

## Abstract

Tuberculous osteomyelitis of the proximal tibia is an extremely rare condition in pediatric patients, often presenting as chronic pain, swelling, and nonspecific symptoms that mimic pyogenic osteomyelitis or bone tumors. We report the case of a three-year-old boy who presented with localized swelling and pain in the right proximal tibia for the past 1.5 months. A plain radiograph revealed lytic lesions with marrow involvement, and a biopsy confirmed granulomatous inflammation with caseous necrosis. The patient was diagnosed with tuberculous osteomyelitis and was started on standard anti-tubercular therapy. Significant clinical and radiological improvement was noted on six-monthly follow-up. This case highlights the importance of maintaining a high index of suspicion for skeletal tuberculosis in endemic regions, even in atypical presentations. Early diagnosis and treatment are critical to prevent complications such as growth disturbances, deformities, and joint involvement, ensuring positive outcomes in pediatric patients.

## Introduction

Skeletal tuberculosis (TB) is an uncommon form of extrapulmonary TB, accounting for 10-20% of all extrapulmonary TB and only 1-2% of overall TB cases [1]. It usually involves the spine (Pott’s disease) and major weight-bearing joints like the hips and knees [2]. Tuberculous osteomyelitis, on the other hand, makes up a small percentage of skeletal TB and is extremely rare in the pediatric population [3]. The involvement of the proximal tibia is rare, making the diagnosis challenging, as the clinical manifestation of tuberculous osteomyelitis often mimics that of pyogenic osteomyelitis, bone tumors, or other chronic bone infections [4].

Hematogenous dissemination from a primary tuberculous lesion, most commonly in the lungs, to the metaphyseal zones of long bones is the predominant pathway in the development of tuberculous osteomyelitis [5]. In children, especially, transphyseal vessels allow bacilli to access the epiphysis, destroying the bone and possibly infiltrating the joint. However, the non-specific clinical symptoms, for example, localized swelling, chronic pain, and infrequent restriction of movement, frequently lead to a delayed diagnosis [1]. Tests, including erythrocyte sedimentation rate (ESR) and C-reactive protein (CRP), might be increased with no specificity, and imaging characteristics, including lytic lesions, bone edema, or periosteal reaction, are shared with other conditions [6]. The gold standard is tissue (bone) biopsy and histopathological examination that demonstrates caseous necrosis and granulomatous inflammation, with Ziehl-Neelsen staining revealing acid-fast bacilli [6].

Literature highlights the importance of early diagnosis and initiation of anti-tubercular therapy (ATT) to prevent progressive bone destruction, growth disturbances, and joint deformities [2]. While surgical intervention may be necessary in some cases for debridement or abscess drainage, medical management with ATT remains the cornerstone of treatment [7]. A high index of suspicion, particularly in endemic regions, combined with a multidisciplinary approach involving various departments, is essential for favorable outcomes.

This case report of a three-year-old boy with proximal tibial (metaphyseal) tuberculous osteomyelitis highlights the diagnostic and therapeutic challenges, emphasizing the critical role of clinical suspicion and timely intervention to prevent complications.

## Case presentation

Initial presentation

**Figure 1:**
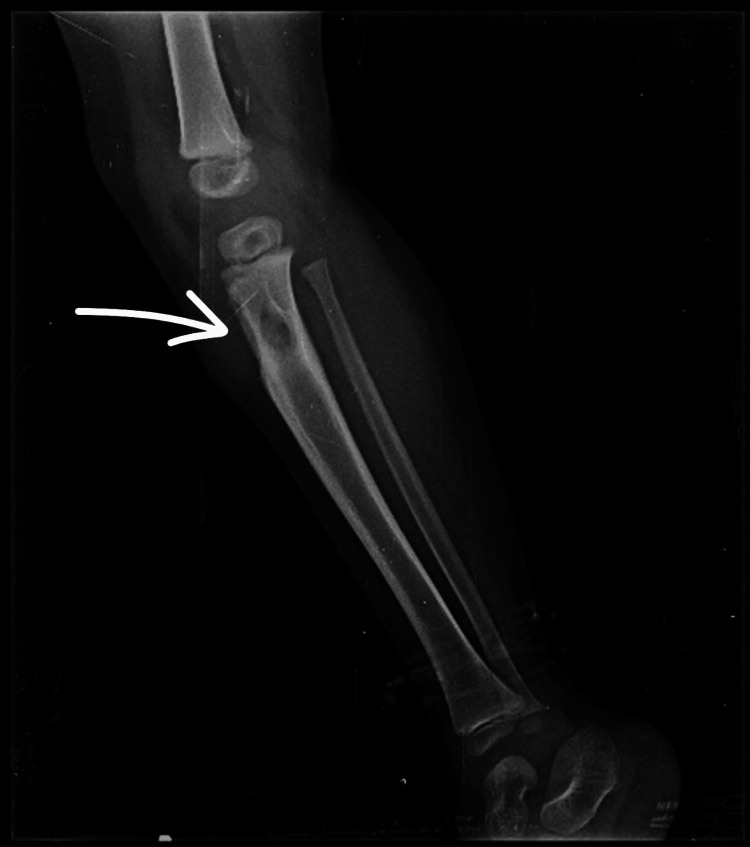
Lateral view of a radiograph of the right leg taken at the time of presentation

**Figure 2:**
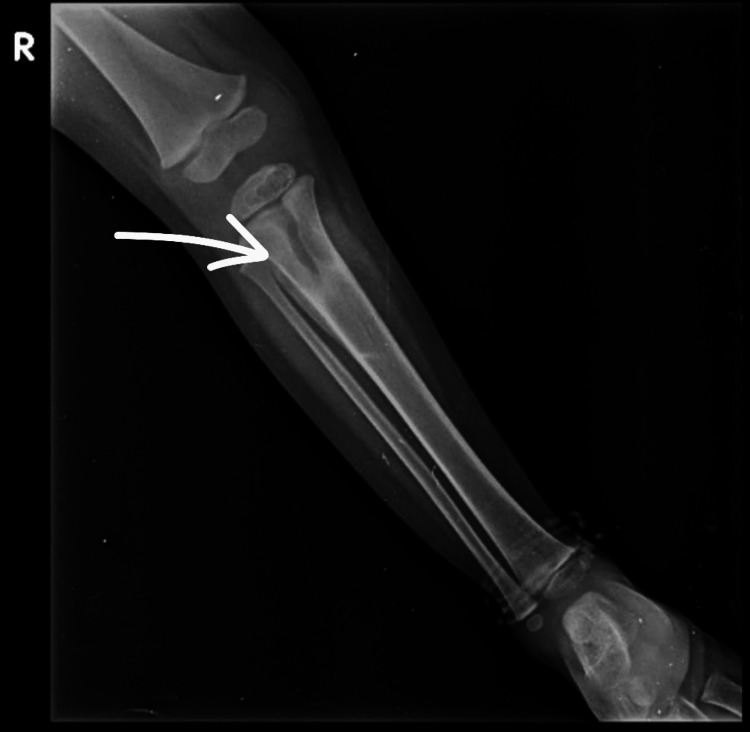
Anteroposterior view of a radiograph of the right leg taken at the time of presentation

A three-year-old boy presented to the outpatient department with swelling over his right proximal leg, pain, difficulty walking, and recurrent fever. He had been asymptomatic until a year ago, when he developed intermittent febrile episodes, swelling, and local warmth over the right proximal leg. Over the past 1.5 months, he experienced a persistent low-grade fever, followed by the onset of pain, swelling, and difficulty walking for the past month. These symptoms progressively worsened over two months. One month prior to the presentation at our hospital, he was evaluated at a local healthcare facility and diagnosed with an active infection over the right proximal leg. He was managed with intravenous antibiotics and incision and drainage. He had an uneventful recovery, regained normal mobility, and remained asymptomatic for months. Two months prior to the current presentation, he developed insidious-onset pain and swelling over the right proximal tibia. There were no aggravating or relieving factors and no trauma. The swelling gradually increased, impairing his ability to walk and perform daily activities. At the time of presentation, the child had a total leukocyte count (TLC) of 11,500 cells/mm³ (normal: 4,000-11,000 cells/mm³), an ESR of 38 mm/hr (normal: <10 mm/hr), and a negative CRP test (normal: <0.9 mg/dL). A plain radiograph showed a cystic lesion involving the metaphyseal and epiphyseal regions of the right proximal tibia (Figures 1-2).

He was brought to our tertiary care medical college hospital, admitted, treated with Linezolid, and discharged. However, symptoms worsened, prompting re-evaluation two weeks later.

Surgical intervention and diagnosis

**Figure 3:**
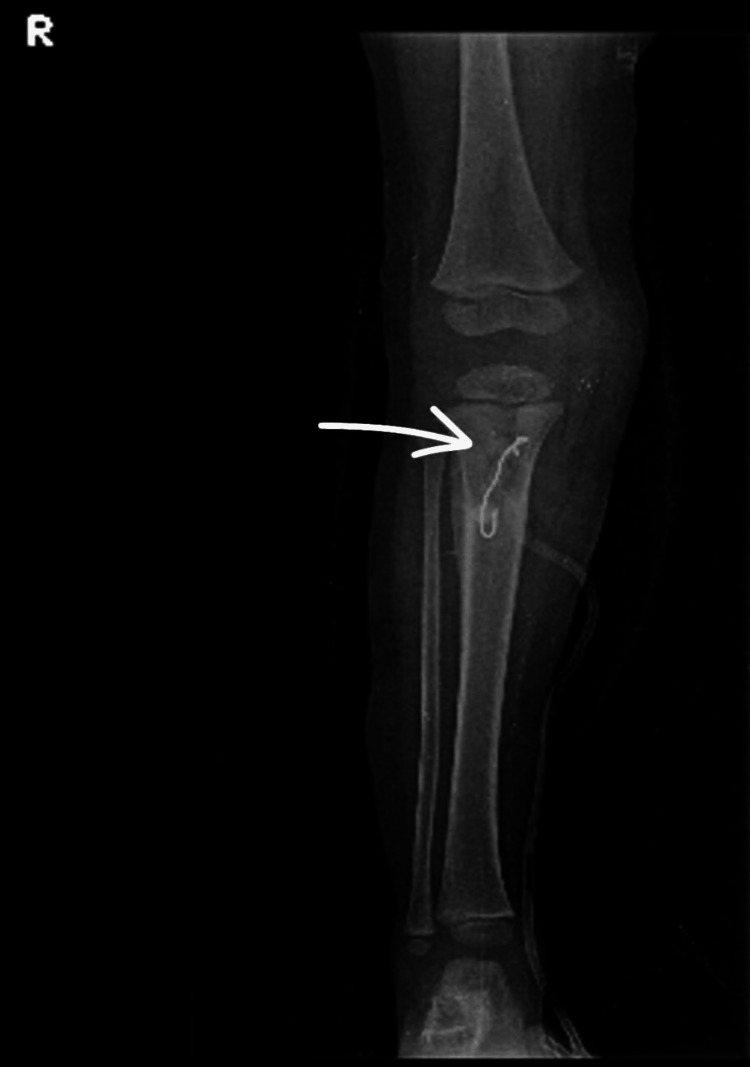
Immediate postoperative anteroposterior radiograph of the right leg showing antibiotic cement bead placement

**Figure 4:**
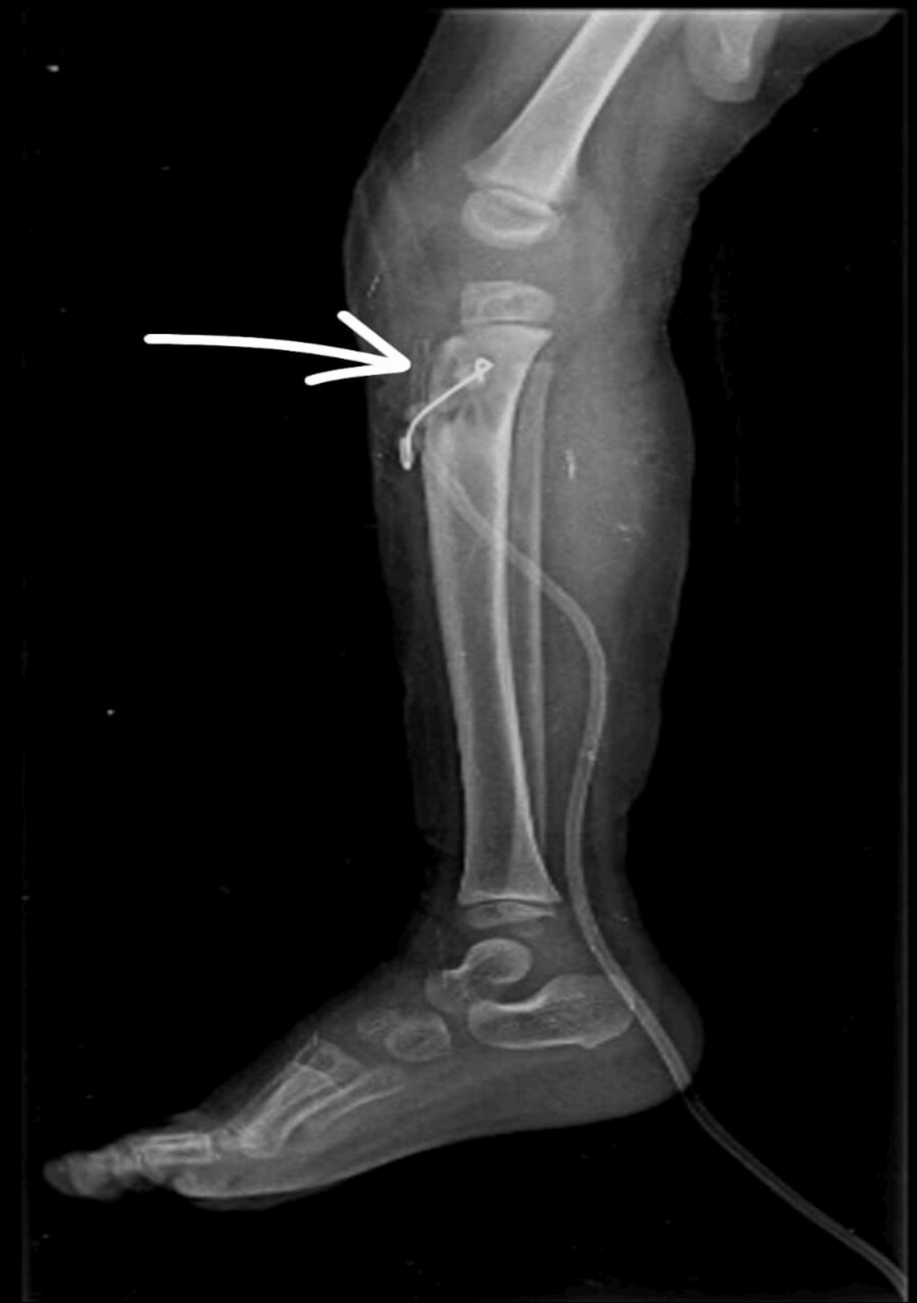
Immediate postoperative lateral view radiograph of the right leg showing antibiotic cement bead placement

The patient underwent emergency wound debridement with curettage and antibiotic cement bead placement (Figures 3-4). Intraoperative tissue, bone biopsy samples, and cultures were sent for analysis. The culture revealed growth of *Escherichia coli*, and the patient was subsequently started on appropriate antibiotics. However, histopathological analysis of the tissue and bone biopsy demonstrated caseous necrosis and granulomatous inflammation, features highly suggestive of tuberculous osteomyelitis. Given the histopathology report, a pulmonology opinion was sought, confirming the suspicion of skeletal TB. The child was initiated on ATT as per standard protocols.

Following the initial surgery, the TLC was 10,800 cells/mm³, ESR was 10 mm/hr, and CRP was negative. Culture and sensitivity (aerobic) of the sample yielded *Escherichia coli*. The organism was resistant to multiple classes of antibiotics, including ciprofloxacin, gentamicin, levofloxacin, tobramycin, ampicillin, and co-trimoxazole, indicating it as an MDR strain. Bone and tissue biopsy revealed bony trabeculae and bone marrow with extensive caseous necrosis, ill-formed granulomas, and multinucleated giant cells.

**Figure 5:**
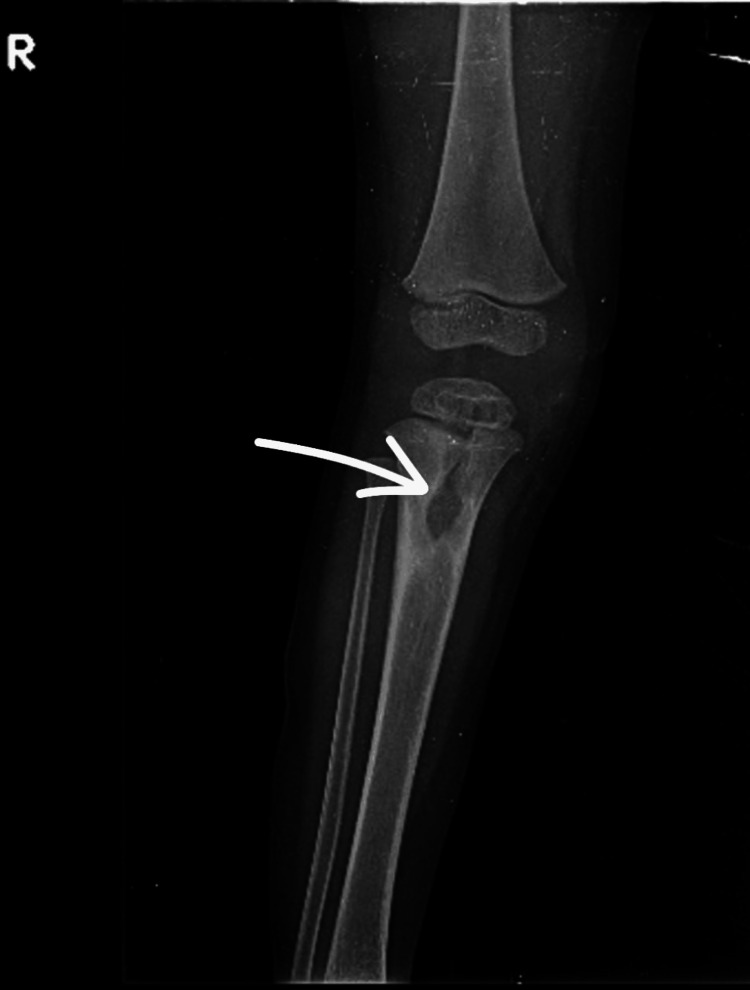
Anteroposterior radiograph of the right knee and leg following removal of antibiotic cement beads

**Figure 6:**
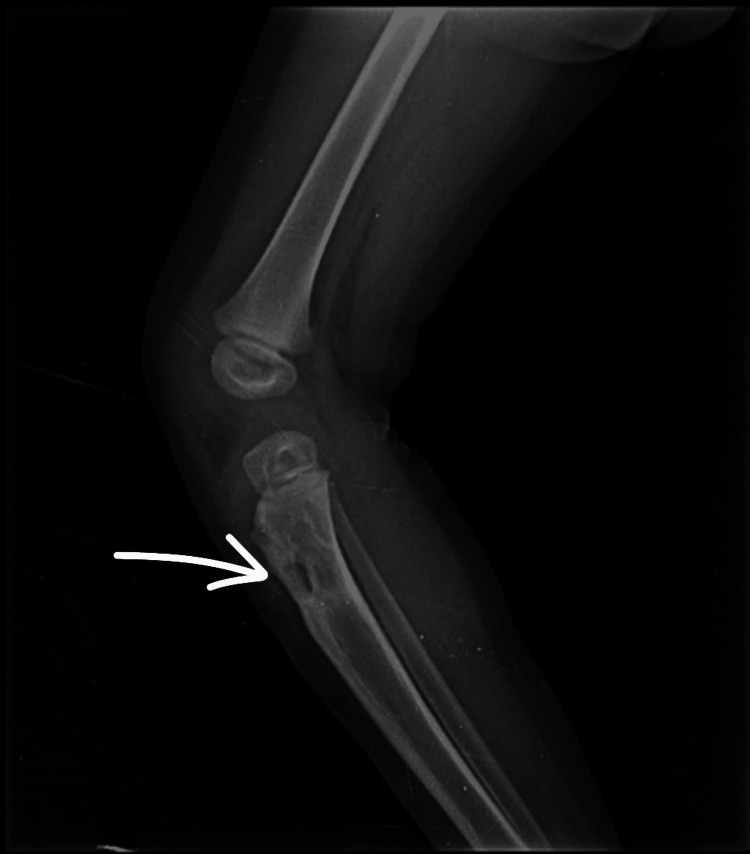
Lateral radiograph of the right knee following removal of antibiotic cement beads

Two months following the surgery, the patient underwent removal of the antibiotic cement beads under short general anesthesia (Figures 5-6). The child continued the full course of ATT for six months as per national TB guidelines, with close monitoring for compliance and side effects. After cement bead removal, the total counts were 10,500 cells/mm³, ESR was 10 mm/hr, and CRP was negative.

Outcome

**Figure 7:**
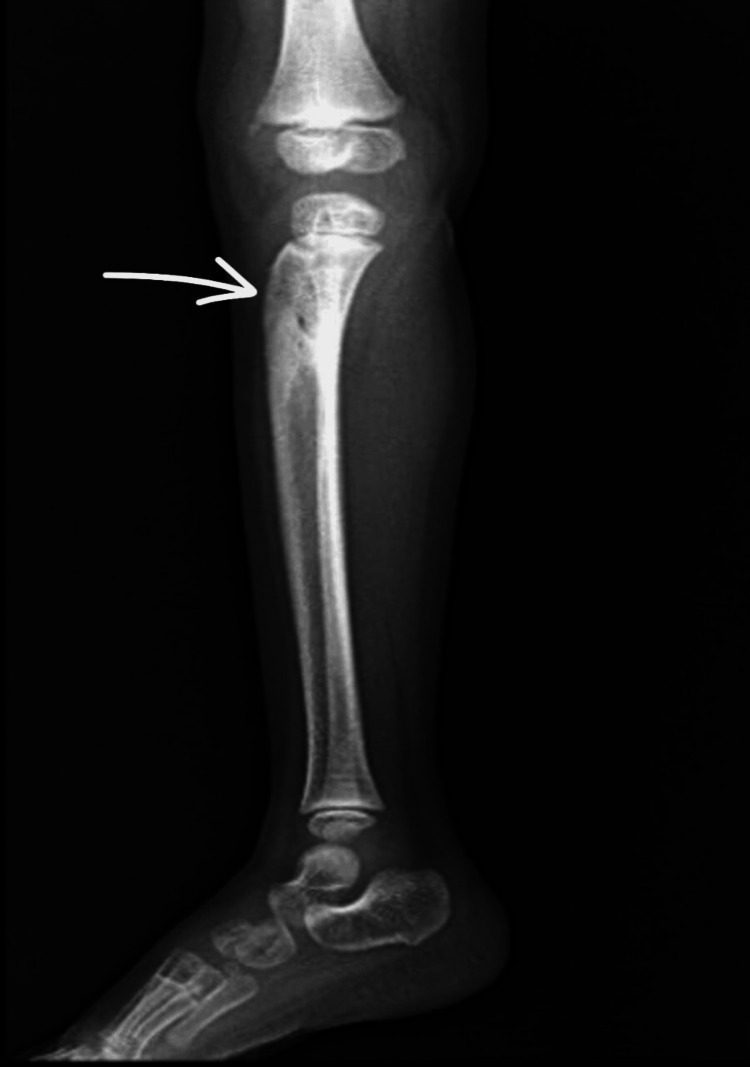
Lateral radiograph of the right leg at six-month follow-up

**Figure 8:**
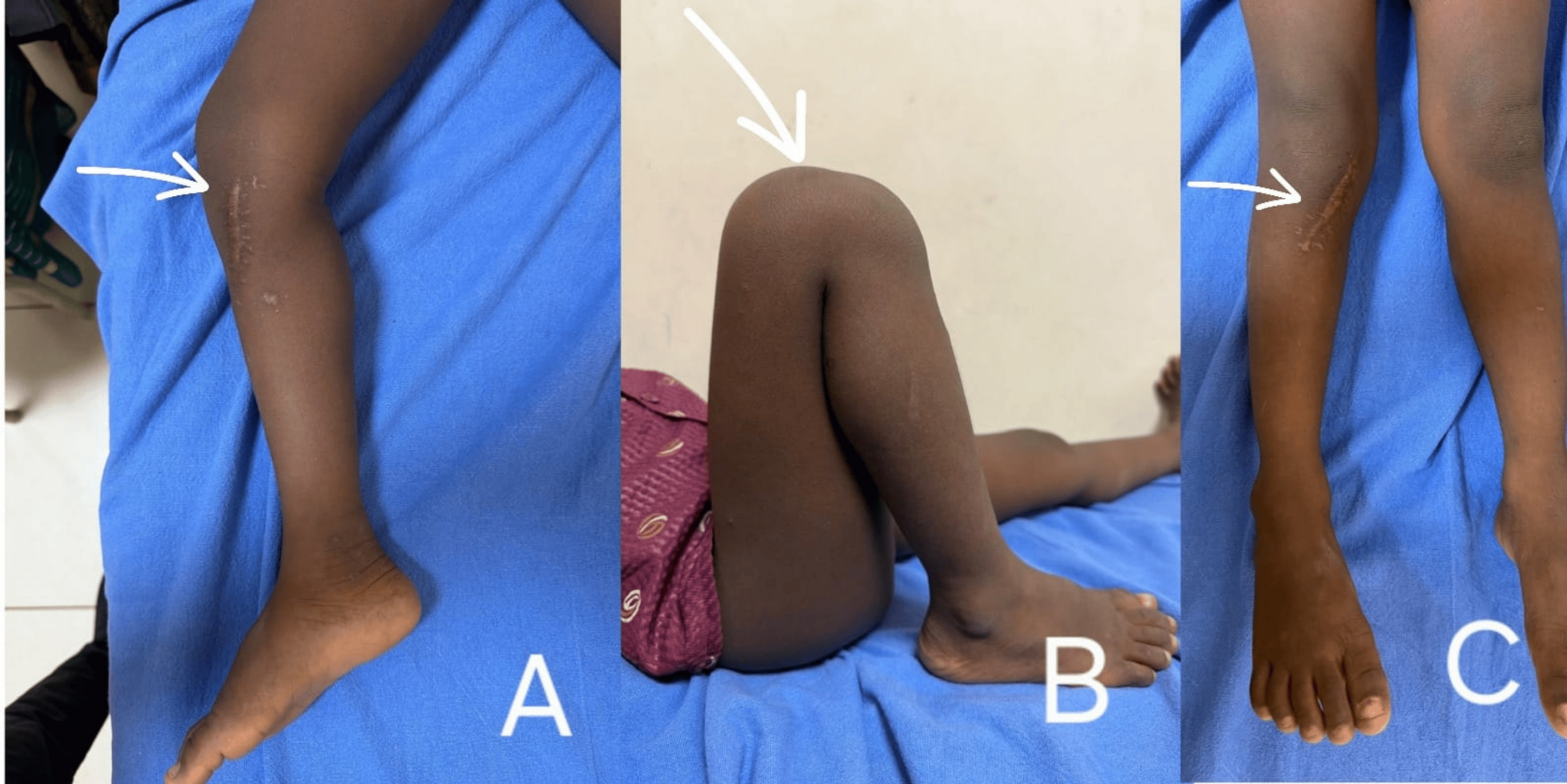
Clinical image at six-month follow-up showing a healthy, healed surgical scar over the right proximal tibia and showing full range of motion of the right knee (A) Knee in full extension with medial aspect of leg displayed. (B) Knee in full flexion with lateral aspect of leg displayed. (C) Clinical image of healthy scar.

At the six-month follow-up, the child demonstrated marked clinical improvement. He was pain-free, with no residual swelling or gait disturbance. The range of motion at the right knee joint was full and unrestricted. Radiological assessment revealed complete healing of the lesion with no evidence of recurrence (Figure 7). The child had to rehabilitate well and has now returned to normal functional activities (Figure 8).

## Discussion

Tuberculous osteomyelitis of the proximal tibia is an uncommon manifestation (1-3%) of skeletal TB, particularly in pediatric populations [8,9]. Skeletal TB accounts for only 1-2% of total TB cases, and the proximal tibia is rarely involved. This rarity, combined with its nonspecific clinical presentation, often delays diagnosis. The symptoms, chronic swelling, localized pain, and restricted movement, closely mimic conditions such as pyogenic osteomyelitis, Brodie’s abscess, or bone tumors, leading to diagnostic challenges [10]. In endemic regions, skeletal TB must be considered in any child presenting with chronic bony lesions and systemic symptoms such as fever [11].

The diagnostic process relies on a combination of clinical suspicion, imaging, histopathology, and microbiological confirmation [12]. Radiological findings often reveal lytic bone lesions with marrow involvement and periosteal reaction, but these are not pathognomonic. Histopathological examination showing caseous necrosis and granulomatous inflammation, along with acid-fast bacilli identification via Ziehl-Neelsen staining, remains the gold standard for diagnosis [4,7]. Molecular tests like GeneXpert MTB/RIF further aid in diagnosis, especially in atypical or challenging cases [7].

In this case, timely recognition and initiation of ATT led to a positive outcome. Early treatment with ATT is critical to prevent complications such as progressive bone destruction, deformities, growth disturbances, and joint involvement, which can severely impact the quality of life [1,4]. Multidisciplinary collaboration between departments is essential for accurate diagnosis and management [13].

This case highlights the importance of maintaining a high index of suspicion for skeletal TB in endemic regions, even in atypical presentations. Pediatric patients with chronic bone symptoms must be thoroughly evaluated for TB, as early diagnosis and treatment significantly improve outcomes. Prompt initiation of ATT can prevent long-term sequelae and ensure favorable recovery, as demonstrated in this case.

## Conclusions

This case emphasizes the importance of considering skeletal TB in the differential diagnosis of chronic osteomyelitis, especially in children from endemic regions. Early diagnosis and prompt initiation of ATT can lead to excellent clinical outcomes and prevent long-term complications. Clinicians should maintain a high index of suspicion for TB in pediatric patients presenting with atypical musculoskeletal symptoms, as in cases of tuberculous osteomyelitis, to facilitate timely diagnosis and appropriate management.
